# Molecular Mechanisms Underlying Abscisic Acid/Gibberellin Balance in the Control of Seed Dormancy and Germination in Cereals

**DOI:** 10.3389/fpls.2018.00668

**Published:** 2018-05-23

**Authors:** Pham A. Tuan, Rohit Kumar, Pawanpuneet K. Rehal, Parneet K. Toora, Belay T. Ayele

**Affiliations:** Department of Plant Science, Faculty of Agricultural and Food Sciences, University of Manitoba, Winnipeg, MB, Canada

**Keywords:** abscisic acid, dormancy, germination, gibberellin, plant hormones, preharvest sprouting, seed, cereals

## Abstract

Seed dormancy is an adaptive trait that does not allow the germination of an intact viable seed under favorable environmental conditions. Non-dormant seeds or seeds with low level of dormancy can germinate readily under optimal environmental conditions, and such a trait leads to preharvest sprouting, germination of seeds on the mother plant prior to harvest, which significantly reduces the yield and quality of cereal crops. High level of dormancy, on the other hand, may lead to non-uniform germination and seedling establishment. Therefore, intermediate dormancy is considered to be a desirable trait as it prevents the problems of sprouting and allows uniformity of postharvest germination of seeds. Induction, maintenance, and release of seed dormancy are complex physiological processes that are influenced by a wide range of endogenous and environmental factors. Plant hormones, mainly abscisic acid (ABA) and gibberellin (GA), are the major endogenous factors that act antagonistically in the control of seed dormancy and germination; ABA positively regulates the induction and maintenance of dormancy, while GA enhances germination. Significant progress has been made in recent years in the elucidation of molecular mechanisms regulating ABA/GA balance and thereby dormancy and germination in cereal seeds, and this review summarizes the current state of knowledge on the topic.

## Introduction

Cereals are among the most economically important crops worldwide with annual production of over 2500 million tons (Food and Agriculture Organization of the United Nations, 2017). However, their production is challenged by a wide range of biotic and abiotic stress factors including the occurrence of high humidity and wet conditions prior to harvest that causes germination of the grain on the spike, which is also referred to as preharvest sprouting. Grain yield and end use quality losses due to preharvest sprouting are reported to cause an annual loss of around $1 billion worldwide (Black et al., 2006). Preharvest sprouting of cereal grains is closely associated with the degree of dormancy, an adaptive trait that inhibits the germination of seeds under optimal environmental conditions (Gao and Ayele, 2014). However, domestication and selection of cereal crops from their wild relatives have been focused on ensuring uniform germination and seedling establishment, and this has led to the development of modern cultivars with low level of dormancy (Lenser and Theißen, 2013; Meyer and Purugganan, 2013; Gao and Ayele, 2014), making the seeds susceptible to field sprouting when moist and wet conditions occur before harvest. Since high level of dormancy in cereal seeds can also cause undesirable consequences such as uneven and slow postharvest germination of seeds, optimum level of seed dormancy is always required to enhance the yield and quality of cereal crops (Gao and Ayele, 2014).

Loss of seed dormancy can be induced by different treatments, including after-ripening, cold stratification, and light (Bewley and Black, 1994). Dormancy loss in the seeds of cereals such as wheat has been shown to be associated with changes in the physiological state of the seed, which involves alterations in gene and protein expression, oxidative modification of gene transcripts and proteins, and epigenetic modifications (Gao et al., 2012, 2013; Gao and Ayele, 2014). Previous studies have also provided important insights into the significance of the metabolic and signaling aspects of different plant hormones and their potential interaction in the maintenance and release of dormancy in cereal seeds (Liu et al., 2013; Chitnis et al., 2014; Shu et al., 2016b). Among the different plant hormones, abscisic acid (ABA) and gibberellin (GA) are considered as major players in the regulation of dormancy and germination; ABA regulates dormancy induction and maintenance positively while GA promotes seed germination (Kucera et al., 2005; Finkelstein et al., 2008). Therefore, changes in the balance of seed ABA/GA levels and sensitivity constitute a central regulatory mechanism underlying the maintenance and release of seed dormancy (Shu et al., 2016b; Finch-Savage and Footitt, 2017). Previous studies have indicated that change in the ABA/GA balance is regulated at least partly by the reciprocal regulation of the expression of genes involved in ABA and GA metabolism and signaling (Seo et al., 2006; Piskurewicz et al., 2008; Izydorczyk et al., 2017). Other endogenous signaling factors such as reactive oxygen species (ROS) and environmental factors such as temperature and light can also influence the balance between ABA and GA, and therefore dormancy and germination in cereal seeds (Gubler et al., 2008; Ishibashi et al., 2015; Izydorczyk et al., 2017).

Recent progress in the genomics of cereal crops has led to the identification of genes involved in ABA and GA metabolism and signaling pathways, and those mediating ABA-GA interactions in cereal crops, and this has opened up new opportunities for elucidating the molecular mechanisms underlying the roles of ABA and GA in the regulation of dormancy and germination in cereal seeds. This review highlights the recent advances made in this regard.

## ABA Regulates the Induction and Maintenance of Seed Dormancy

### ABA Metabolism

**FIGURE 1:**
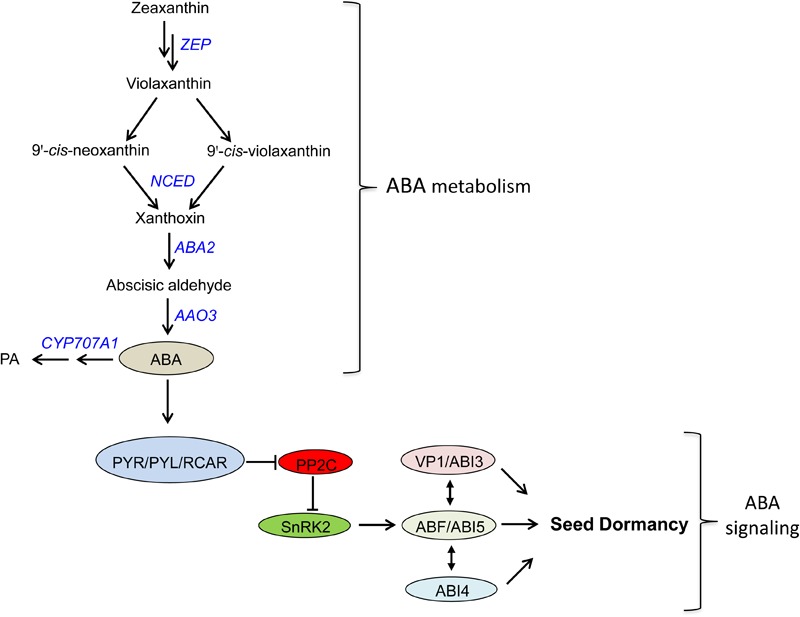
Abscisic acid metabolism and signaling pathway in plants. ZEP, zeaxanthin epoxidase; NCED, 9-*cis*-epoxycarotenoid dioxygenase; ABA2, ABA deficient 2 (short chain alcohol dehydrogenase); AAO3, abscisic aldehyde oxidase; CYP707A1, a cytochrome P450 monooxygenase gene encoding ABA-8′-hydroxylases; PA, phaseic acid; PYR/PYL/RCAR, pyrabactin resistance/pyrabactin-like/regulatory components of ABA receptors; PP2C, protein phosphatase 2C; SnRK2, SNF1-related protein kinase2; ABI3, abscisic acid insensitive 3; ABI4, abscisic acid insensitive 4; ABI5, abscisic acid insensitive 5; VP1, viviparous 1; ABF, ABRE binding factor.

Abscisic acid is one of the major plant hormones involved in many aspects of plant growth and developmental processes including seed dormancy and germination. The level of ABA in plant tissues/seeds is regulated by its biosynthesis and catabolism (Nambara and Marion-Poll, 2005; Nambara et al., 2010). Of the several steps involved in the biosynthesis of ABA, oxidative cleavage of 9-*cis*-neoxanthin and violaxanthin by 9-*cis*-epoxycarotenoid dioxygenase (NCED) is reported to be the rate-limiting step (Schwartz et al., 2003) while ABA catabolism mainly takes place through hydroxylation of ABA at the 8′ position by the action of ABA 8′-hydroxylase (ABA8′OH) (Cutler and Krochko, 1999), which is encoded by *CYP707A* genes (Kushiro et al., 2004; Saito et al., 2004) (**Figure 1**). Therefore, the expressions of *NCED* and *CYP707A* genes play significant roles in the control of seed ABA level, and therefore dormancy and germination.

#### ABA Metabolism and Its Role in Regulating Dormancy During Seed Development in Cereals

Seed development in dicot species such as Arabidopsis is characterized by two peaks of ABA accumulation that occur during the mid- and late-phases of seed maturation; ABA in the latter peak, which is mainly synthesized in the zygotic tissues (Kanno et al., 2010), plays a key role in the induction and maintenance of seed dormancy (Kermode, 2005; Nambara et al., 2010). Similarly, two peaks of embryonic ABA level have been detected during wheat seed development (King, 1976; Suzuki et al., 2000). The first peak of ABA occurs at about 25 days after pollination (DAP) while the second peak occurs at about 35 DAP. However, the second ABA peak appears to occur for an extended duration, that is, up to 40 DAP in the seeds of dormant wheat line as compared to the non-dormant line (Suzuki et al., 2000), suggesting the involvement of ABA synthesized during the late phase of seed maturation for the establishment of seed dormancy. Furthermore, maintenance of elevated embryonic ABA level for prolonged duration during the late-maturation phase of wheat seed development has been reported to be associated with the higher level of dormancy (Walker-Simmons and Sesing, 1990) while maturing seeds (at about 30 DAP) of a non-dormant mutant line of wheat have been shown to exhibit a significantly lower level of embryonic ABA than that observed in the dormant line from which the mutant is generated (Kawakami et al., 1997). In agreement with this, mutations in the two homolog of *TaABA8*′*OH1* (*TaABA8*′*OH1A* and *TaABA8*′*OH1D*) that cause an increase in embryonic ABA content during the middle and later stages of seed development (40–60 DAA) have been shown to result in an enhanced degree of seed dormancy (Chono et al., 2013), reinforcing the significance of elevated embryonic ABA level during the maturation phase of seed development in the induction of dormancy in wheat seeds. Although these studies provided important insights into the role of ABA in dormancy induction in wheat seeds, detailed understanding of the molecular mechanisms underlying the regulation of ABA metabolism during seed maturation and therefore dormancy induction awaits further study.

A study in rice seeds, however, implicated that the accumulation of ABA involved in the induction of dormancy occurs at the earlier stage of seed development; rice cultivars with deep dormancy exhibit higher seed ABA level during the early and middle stage of seed development (10–20 DAP) than that observed in cultivars with medium and low levels of dormancy (Liu et al., 2014). In addition, ABA accumulation during seed development in weedy red rice has been shown to peak at 10 DAP; however, seeds of dormant lines contain over twofold more ABA than that observed in non-dormant lines and this variation in seed ABA content appeared to be associated at least partly with the expression patterns of *NCED1* paralogs (Gu et al., 2011). In barley, developing seeds of both dormant and non-dormant cultivars have been shown to exhibit a high amount of embryonic ABA through the physiological maturity phase after which embryonic ABA level declined substantially in the non-dormant but not in the dormant cultivar (Benech-Arnold et al., 1999). Analysis of the expression patterns of ABA metabolic genes in developing barley seeds revealed that *HvNCED2* regulates ABA level during the early- to mid-phases of seed maturation while *HvABA8*′*OH1* (a barley homolog of *CYP707A*) is responsible for controlling seed ABA level thereafter (Chono et al., 2006). These results suggest the importance of ABA catabolism in controlling seed ABA level and dormancy induction in maturing barley seeds. Furthermore, seed development in a triticale cultivar that is less susceptible to preharvest sprouting has been shown to be characterized by higher *TsNCED1* expression and ABA level than that observed in a cultivar that is more sensitive to preharvest sprouting, suggesting the role of ABA biosynthesis in the regulation of dormancy establishment in cereal seeds (Fidler et al., 2016).

#### ABA Metabolism and Its Role in Regulating Seed Dormancy Maintenance and Loss in Cereals

Seed dormancy is partly determined by the level of ABA contained in mature seeds (Finkelstein et al., 2008; Nambara et al., 2010). Previous studies have shown that dormancy loss and germination of cereal seeds are associated with a decrease in seed ABA level during imbibition, and the change in seed ABA level appears to be related with the expression patterns of *NCED* and *CYP707A* genes. For example, after-ripening induces loss of seed dormancy in barley, and this is mediated by imbibition-induced reduction in seed ABA content to a level lower than that observed in the corresponding dormant seeds. This difference in ABA content and therefore level of dormancy is reported to be associated mainly with the expression pattern of *HvABA8*′*OH1* (Millar et al., 2006; Gubler et al., 2008). Consistently, RNAi-mediated knock-down of *HvABA8*′*OH1* leads to enhanced seed ABA level and dormancy (Gubler et al., 2008). Studies in rice also indicated that the reduction of ABA level during imbibition of non-dormant seeds of rice is related mainly with increased expression of *OsABA8ox* (rice homologs of *CYP707A*) genes (Zhu et al., 2009; Du et al., 2015). These results suggest the significance of ABA catabolism in the regulation of ABA level and seed dormancy maintenance and release in the seeds of cereals such as rice and barley.

Other studies in barley and *Brachypodium* have also demonstrated the importance of ABA biosynthesis and/or catabolism in the regulation of seed dormancy. For example, after-ripening of dormant *Brachypodium* seeds leads to a decrease in the expression of *BdNCED1* along with a transient induction in the expression of *BdABA8*′*OH1* (a *Brachypodium* homolog of *CYP707A*) during imbibition, resulting in a reduced ABA level and enhanced seed germination (Barrero et al., 2012). Furthermore, imbibition of dormant seeds of barley under white light is reported to enhance the expression of *HvNCED1* with no marked effect on that of *HvABA8*′*OH1*, leading to increased embryonic ABA content and inhibition of seed germination (Gubler et al., 2008). White light has also been reported to promote dormancy in both dormant and non-dormant seeds of *Brachypodium*, although this effect does not appear to be closely associated with changes in ABA metabolism/seed ABA level (Barrero et al., 2012). Exposure of partially after-ripened barley seeds to blue light has also been shown to induce the expression of *HvNCED1* and repress that of *HvABA8*′*OH1* in the embryo, resulting in an increase in embryonic ABA level and suppression of germination (Barrero et al., 2014). Blue light also induces secondary dormancy in barley seeds, and this appears to be associated with an increase in embryonic ABA content, which is mediated by enhanced expression of *HvNCED1* and *HvNCED2*, and decreased expression of *HvABA8*′*OH1* (Hoang et al., 2014). Recent progress in the molecular aspects of ABA metabolism and dormancy regulation in wheat seeds is highlighted in the following section.

Similar to that observed in the seeds of other cereal crops, after-ripening of dormant wheat seeds leads to imbibition-mediated reduction of ABA level, while maintenance of elevated ABA level was evident in the corresponding dormant seeds, and these differences in ABA level are mediated by changes in the expression patterns of *TaNCED1* and *TaABA8*′*OH1* (a wheat homolog of *CYP707A*) genes (Jacobsen et al., 2013), implying the contribution of both ABA biosynthesis and catabolism in the regulation of seed ABA level and dormancy in wheat. Furthermore, seeds from a dormant wheat genotype have been shown to exhibit higher and lower expression of *TaNCED2* and *TaABA8*′*OH1*, respectively, in both dry and imbibed states than those derived from a non-dormant genotype (Son et al., 2016). Consistently, ectopic expression of *TaNCED2A* (a homeolog of *TaNCED2* from the A genome of wheat) and *TaABA8*′*OH1B* (a homeolog of *TaABA8*′*OH1* from the B genome of wheat) in Arabidopsis caused alteration of seed ABA level and dormancy (Son et al., 2016), and a double mutation in the A and D genome copies of *TaABA8*′*OH1* resulted in an increase in embryonic ABA level and a decrease in seed germination (Chono et al., 2013). Other treatments that lead to dormancy loss in wheat seeds also affect the expression patterns of ABA metabolic genes and ABA level. For example, dormancy loss due to imbibition of wheat seeds at lower temperature (15°C) decreases ABA level in the embryos via increased expression of *TaABA8*′*OH*s (Kashiwakura et al., 2016). Furthermore, after-ripening and cold stratification mediated dormancy loss in wheat seeds is reported to be associated with decreased ABA levels in the embryos and aleurone (Tuttle et al., 2015). Moreover, the effect of different seed imbibition temperature regimes on wheat seed germination is reported to be mediated by changes in the expressions of *TaNCED* and *TaABA8*′*OH* genes, and seed ABA level (Izydorczyk et al., 2017). For example, delay in the germination of wheat seeds due to imbibition under supraoptimal temperature (35°C) is associated with elevated embryonic ABA level, which is mediated via enhanced expression of *TaNCED*s; whereas inhibition of germination by suboptimal temperature (4°C) appears to be caused by increased ABA level in both embryo and endosperm tissues, which is regulated by increased and decreased expression of *TaNCED*s and *TaABA8*′*OH*s, respectively, in both tissues (Izydorczyk et al., 2017).

### ABA Signaling

ABA signaling involves three major core components; PYRABACTIN RESISTANCE1/PYRABACTIN-LIKE/REGULATORY COMPONENTS OF ABA RECEPTORS (PYR/PYL/RCAR), PROTEIN PHOSPHATASE 2Cs (PP2Cs; negative regulators), and SNF1-RELATED PROTEIN KINASE 2s (SnRK2s; positive regulators) (**Figure 1**). In the absence of ABA, PP2Cs inhibits the activities of SnRK2s through dephosphorylation of their kinase activation loop; however, when ABA is present, the ABA receptors PYR/PYL/RCAR form a complex with PP2C, and this inhibits the phosphatase activity of PP2C and thereby activate SnRK2 (Finkelstein, 2013; Ng et al., 2014). The activated form of SnRK2 subsequently turns on ABRE-binding protein/ABRE-binding factor (AREB/ABF) transcription factors, which in turn activates the transcription of ABA responsive genes (Ng et al., 2014). Among AREB/ABF transcription factors, ABA insensitive 5 (ABI5), a member of the basic leucine zipper transcription factor family, plays a central role in regulating ABA-responsive genes in seeds (Nambara et al., 2010; Finkelstein, 2013). In addition, ABI4 and ABI3, AP2-type and B3-type transcription factors, respectively, have been reported to function along with ABI5 to induce the expression of ABA responsive genes, and thereby regulate seed dormancy and germination (**Figure 1**).

#### ABA Signaling and Its Role in Regulating Seed Dormancy Maintenance and Loss in Cereals

Dormancy maintenance in cereal seeds is also determined by seed sensitivity to ABA (Walker-Simmons, 1987; Steinbach et al., 1995; Tuttle et al., 2015), which is regulated by the expression of genes involved in the ABA signaling pathway including those encoding the three central components (PYR/PYL/RCAR5, PP2C, and SnRK2) and the downstream transcription factors including ABI3, ABI4, and ABI5 (Nambara et al., 2010). Previous studies have demonstrated that mutants of *ABI3, ABI4*, and *ABI5* exhibit ABA resistant germination phenotype, and these transcription factors appear to interact extensively (Söderman et al., 2000) and control the expression of ABA responsive genes that are involved in the regulation of seed dormancy/germination, including those involved in ABA catabolism such as *CYP707A1* and *CYP707A2* (Shu et al., 2013), GA catabolism such as *GA2ox3* (Cantoro et al., 2013), and starch degradation such as α*-amylase* (Hoecker et al., 1995) genes. One of the first key ABA signaling components identified and functionally characterized with respect to seed dormancy in cereals is the maize *Viviparous1* (*Vp1*) gene, an ortholog of the *ABI3* of Arabidopsis (McCarty et al., 1991). Embryos of maize seeds that exhibit varying degree of resistance to preharvest sprouting have been reported to exhibit temporal difference in the expression of *Vp1* during embryogenesis (Carrari et al., 2001), suggesting its role in the control of dormancy. Consistently, mutations in *Vp1* lead to disruption of embryo maturation and induction of its germination while still on the cob (McCarty et al., 1991; Hoecker et al., 1995). On the other hand, ectopic expression of the maize *Vp1* gene in wheat results in increased level of seed dormancy and tolerance to preharvest sprouting (Huang et al., 2012). It has been shown previously in pea that alternative splicing of the *ABI3* homolog results in the formation of non-functional/truncated protein products from mis-spliced transcripts (Gagete et al., 2009), and such an event has been observed in rice and wheat genotypes exhibiting early dormancy release (McKibbin et al., 2002; Fan et al., 2007). Furthermore, the level of dormancy in wheat seeds has been shown to be associated with the expression pattern of *Vp1* (Nakamura and Toyama, 2001; Laethauwer et al., 2012).

Previous reports have also implicated other ABA signaling components in the regulation of dormancy and germination in cereal seeds. For example, seeds of rice plants overexpressing *PYL*/*RCAR5* exhibit hypersensitivity to ABA and delayed germination phenotype (Kim et al., 2012). Moreover, immature seeds (30 DAP, before physiological maturity) from a dormant line of sorghum exhibit transcriptional induction of *PKABA1* (*SnRK2* ortholog), *ABI3, ABI4*, and *ABI5*, and enhanced expression of ABI5 protein during imbibition as compared to those derived from the less-dormant line (Rodríguez et al., 2009). A recent transcriptomic analysis of maturing seeds from dormant and non-dormant genotypes of wheat revealed the enrichment of several ABRE motifs, which act as binding sites for ABI5, in the embryo and endosperm gene co-expression clusters of both genotypes, of which G-box-like motif is specifically enriched in both tissues of seeds derived from the dormant genotype, suggesting importance of ABI5 in the control dormancy establishment is mediated by specific ABRE motifs (Yamasaki et al., 2017). Increased expression of *TaABF1*, a wheat ortholog of *ABI5*, has also been reported during imbibition of dormant wheat grains (Johnson et al., 2002). On the other hand, dormancy release due to after-ripening in wheat and barley seeds is associated with transcriptional repression of *SnRK2, ABI5*, and *ABI3-interacting protein2* (*AIP2*) during imbibition (Barrero et al., 2009; Liu et al., 2013), suggesting a decrease in seed ABA sensitivity. A recent study has also elucidated the role of ABI4 in regulating ABA sensitivity and thereby dormancy and germination in Arabidopsis seed (Shu et al., 2013); however, the physiological role of this ABA signaling components has yet to be determined in cereal seeds.

Environmental factors such as temperature also modulate the expression of ABA signaling genes and thereby ABA sensitivity of wheat seeds during imbibition. It has been shown recently that imbibition of non-dormant wheat seeds under supraoptimal temperature leads to delay in germination via inducing the expression of *PYL5, SnRK2, ABI3*, and *ABI5* genes in the embryo tissue and thereby enhancing its sensitivity to ABA. Similarly, the inhibitory effect of suboptimal imbibition temperature on germination appears to be mediated by increased ABA sensitivity of both embryo and endosperm tissues through transcriptional activation of the ABA signaling genes; *PYL5, SnRK2, ABI3*, and *ABI5* in the embryo, and *SnRK2* and *ABI5* in the endosperm (Izydorczyk et al., 2017).

## Gibberellin Regulates Seed Dormancy and Germination in Cereals

### Gibberellin Metabolism

**FIGURE 2:**
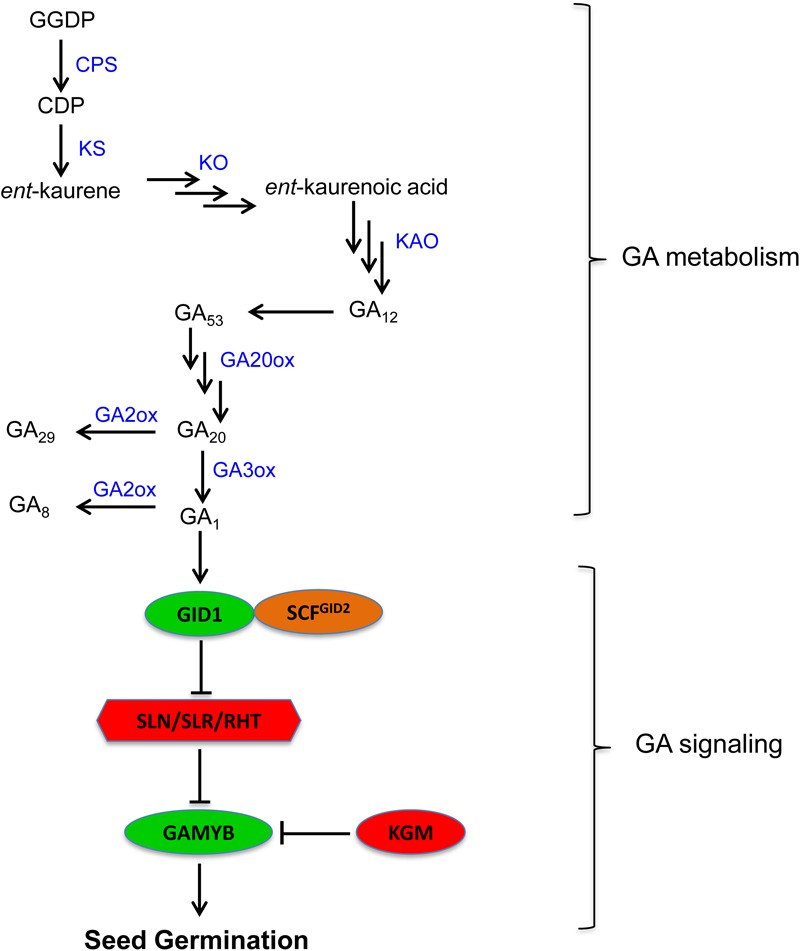
Gibberellin metabolism and signaling pathway in plants (GA_1_ is the major bioactive GA in seeds of cereals such as wheat). GGDP, geranylgeranyl diphosphate; CDP, *ent*-copalyl diphosphate; CPS, *ent*-copalyl diphosphate synthase; KS, *ent*-kaurene synthase; KO, *ent*-kaurene oxidase; KAO, *ent*-kaurenoic acid oxidase; GA20ox, GA20 oxidase; GA3ox, GA3 oxidase; GA2ox, GA2 oxidase; GID1, gibberellin insensitive dwarf 1; GID2, gibberellin insensitive dwarf 2; SLN, slender1 in barley; SLR1, slender rice1; RHT, reduced height; GAMYB, GA regulated MYB transcriptional regulator; KGM, kinase associated with GAMYB.

Gibberellin is the other major phytohormone with an important role in the regulation of seed dormancy and germination (Finch-Savage and Leubner-Metzger, 2006). The level of biologically active GAs in plant tissues is determined by the balance between its biosynthesis and inactivation (Yamaguchi, 2008). The biosynthesis of GA is regulated mainly by reactions catalyzed by GA 20-oxidase (GA20ox) and GA 3-oxidase (GA3ox) while its inactivation is controlled primarily by GA 2-oxidase (GA2ox) (**Figure 2**). Genes encoding these enzymes have been identified from several crop species including cereals such as rice, barley, and wheat (Spielmeyer et al., 2004; Yamaguchi, 2008; Pearce et al., 2015), and their expression play important roles in regulating seed GA level, and therefore dormancy and germination.

#### Gibberellin Metabolism and Its Role in Regulating Seed Dormancy and Germination in Cereals

Several genetic and physiological analyses of the seeds of dicot species such as Arabidopsis and tomato have demonstrated the requirement of GA for germination; GA promotes germination through enhancing the growth potential of the embryo and overcoming the mechanical barriers imposed by covering layers surrounding embryo (Debeaujon and Koornneef, 2000; Kucera et al., 2005). With respect to cereals, reports that implicate GA in the control of seed dormancy and germination are mainly based on comparative analysis of dormant and non-dormant seeds. For example, dormancy loss in wheat and barley seeds due to after-ripening has been shown to be associated with enhanced expression of the *TaGA20ox* and *TaGA3ox* genes and increased level of bioactive GA_1_ during imbibition (Gubler et al., 2008; Liu et al., 2013; Kashiwakura et al., 2016). Consistently, comparative genomic analysis among barley, wheat, and rice identified *HvGA20ox* as a candidate gene regulating a seed dormancy QTL in barley (Li et al., 2004). Likewise, the expression of the GA biosynthetic genes (*GA20ox* and *GA3ox2*) is induced while that of GA inactivating genes (*GA2ox*) is repressed during imbibition of non-dormant rice and sorghum seeds, and this has been shown to be associated with increased level of bioactive GA (Kaneko et al., 2002; Rodríguez et al., 2012; Du et al., 2015). Recent genetic studies in rice have identified *OsGA20ox2* and *OsGA2ox3* as candidate genes for controlling seed germination (Ye et al., 2015; Magwa et al., 2016), and loss of function mutation in *OsGA20ox2* leads to reduced seed GA level and enhanced dormancy (Ye et al., 2015).

Exogenous factors that are implicated in the regulation of dormancy in cereal seeds such as temperature can also induce changes in the expression patterns of GA metabolic genes. For example, dormancy decay in wheat seeds due to imbibition at lower than optimal temperature (15°C) is related with enhanced expression of *TaGA3ox2* and thereby increased level of bioactive GA in the embryo (Kashiwakura et al., 2016) while inhibition of germination due to imbibition of non-dormant wheat seeds at suboptimal temperature (4°C) is related with decreased expression of *TaGA20ox*s and *TaGA3ox2*, and reduced bioactive GA level in both embryo and endosperm tissues (Izydorczyk et al., 2017). However, further genetic analyses are required for detailed understanding of the physiological roles of GA metabolic genes in the regulation of seed dormancy in cereals.

### Gibberellin Signaling

Gibberellin signaling in plants is triggered when bioactive GA is perceived by its receptor GIBBERELLIN INSENSITIVE DWARF1 (GID1) (Ueguchi-Tanaka et al., 2005) (**Figure 2**). The prevalence of GA responses such as seed germination requires GA induced degradation of DELLA protein, which acts as a negative regulator of GA signaling (Sun, 2011). The binding of GA to GID1 promotes the formation of GA-GID1-DELLA complex (Murase et al., 2008), which in turn associates with F-box protein, the central component of SCF^SLY 1/GID2^ E3 ubiquitin ligase, leading to DELLA degradation via the ubiquitin-26S proteasome pathway (McGinnis et al., 2003; Sasaki et al., 2003). The degradation of DELLA by GA activates GAMYB, a downstream GA signaling component that mediates GA signaling effects (Gubler et al., 1995, 1999). The function of GAMYB in cereal aleurone cells has been reported to be repressed by another downstream GA signaling component designated as KINASE ASSOCIATED WITH GAMYB1 (KGM1) (Woodger et al., 2003).

#### Gibberellin Signaling and Its Role in Regulating Seed Dormancy and Germination in Cereals

Seed dormancy or germination in cereals such as wheat is also reported to be associated with changes in seed sensitivity to GA (Tuttle et al., 2015), which is influenced by the expression of genes encoding the GA signaling components. The gene encoding GID1 was first identified from rice and later in other cereals such as barley (Chandler et al., 2008) and wheat (Li et al., 2013). Genetic analysis of rice mutant lines showed that GA responses are mediated by GID1 in the nucleus of aleurone cells and there is no alternative GA receptor in the rice aleurone (Yano et al., 2015). However, mutation in *GID1* of rice does not appear to affect seed germination although it causes repression of the activity of α-amylase (Ueguchi-Tanaka et al., 2005). Furthermore, the expression pattern of *GID1* is found not to be associated with the dormancy or germination phenotype of wheat and barley seeds (Barrero et al., 2009; Liu et al., 2013; Izydorczyk et al., 2017). Studies in rice and wheat have shown that GID2 is a part of SCF complex and positively regulates the GA response (Hirano et al., 2010; Lou et al., 2016); however, mutation in GID2 of rice leads to repression of the α-amylase activity with no any effect on seed germination (Ueguchi-Tanaka et al., 2005). Similar to that observed for *GID1*, the expression of *GID2* is not associated with the seed germination phenotype (Ueguchi-Tanaka et al., 2008). These results overall suggest that GA signaling in the seeds of cereals such as wheat, barley, and rice can operate independent of GID1 and GID2 levels; however, more studies are required to validate these observations.

Cereals appear to have a single or fewer DELLA proteins that are reported to be highly conserved (Davière and Achard, 2013) including SLENDER RICE1 (SLR1) of rice (Ikeda et al., 2001), SLENDER1 (SLN1) of barley (Chandler et al., 2002), REDUCED HEIGHT (RHT) of wheat (Peng et al., 1999), and D8 and D9 of maize (Lawit et al., 2010). Overexpression of *SLN1* leads to repression of GA induced α*-amylase* expression in barley seeds (Zentella et al., 2002), and in agreement with this, the *SLN* mutant of barley is characterized by non-dormant seeds with high amylase activity in the aleurone (Chandler, 1988). A recent report in wheat showed that inhibition of the germination of non-dormant wheat seeds due to imbibition at suboptimal and supraoptimal temperatures is associated with increased expression of *RHT1*, suggesting the role of GA signaling or seed GA sensitivity in the induction of dormancy (Izydorczyk et al., 2017). Another evidence for the role of *RHT1* in seed dormancy and germination comes from the identification of *RHT1* alleles that are able to produce different levels of seed dormancy (Velde et al., 2017). In contrast, no differential expression of *RHT* was observed during imbibition of dormant and after-ripened seeds of wheat (Liu et al., 2013); however, this discrepancy could be due to the fact that the whole seed tissue instead seed embryo was analyzed in this particular study.

The degradation of DELLA (SLN/SLR) by GA activates *GAMYB*, which in turn induces the transcription of α*-amylase* in the aleurone of barley and rice seeds via binding to the GA-responsive elements (GARE) present in its promoter (Gubler et al., 1995, 1999; Sun and Gubler, 2004). Furthermore, GAMYB has been shown to interact synergistically with other transcription factors such as DNA binding with one finger (DOF) proteins and regulate the expression of α*-amylase* in barley (Zou et al., 2008). On the other hand, GAMYB regulates other transcription factors such as GATA type transcription factors that act as positive regulators of seed dormancy (Ravindran et al., 2017), and transcription factors such as KGM regulates *GAMYB* negatively and thereby lead to repression of genes encoding hydrolases in the aleurone (Woodger et al., 2003). Despite their reported functionalities, no differential expression of *GAMYB* and *KGM1* was evident between dormant and after ripened/non-dormant seeds of wheat (Liu et al., 2013). The induction in the expression of hydrolases during imbibition of after-ripened wheat seeds irrespective of the absence of differential expression of *GAMYB* and *KGM* between the dormant and after-ripened seed samples might suggest that GA signaling in cereal seeds is not dependent on the level of the two transcriptional regulators. Functional analysis of *GAMYB* with respect to dormancy, and identification and characterization of other molecular features that regulate or interact with GAMYB are crucial to enhance our understanding of the role of downstream GA signaling elements in the control of seed dormancy and germination in cereals.

## Abscisic Acid/Gibberellin Balance Controls Seed Dormancy and Germination in Cereals

### Endogenous Factors Involved in the Regulation of ABA/GA Balance in Cereal Seeds

Several studies have indicated that the dynamic balance between ABA and GA metabolism and thereby the developmental switch between dormancy and germination can be modulated by their reciprocal regulation (Seo et al., 2006; Piskurewicz et al., 2008; Izydorczyk et al., 2017). It has been shown previously that vivipary phenotype in maize kernels due to ABA deficiency can be reversed through inhibition of GA synthesis, and this demonstrates the role of GA in antagonizing the action of ABA and thereby seed transition from dormancy to germination (White et al., 2000). Changes in the balance between ABA and GA levels, which modulate seed dormancy status, are associated with alterations in the expression patterns of their metabolic genes; for example, imbibition of non-dormant barley seeds induces the expression of ABA catabolic gene *HvABA8′OH1* and GA biosynthetic gene *HvGA3ox2* (Suzuki et al., 2005; Millar et al., 2006), which leads to reduced ABA and increased GA levels. Furthermore, imbibition of non-dormant wheat seeds has been shown to lead to transcriptional induction of *TaGA3ox2* and *HvABA8′OH1* genes (Liu et al., 2013; Son et al., 2016). Further genetic studies such as mutational and gain of function studies are required to precisely elucidate the antagonistic relationship between ABA and GA metabolic pathways in regulating the switch of cereal seeds between dormancy and germination.

In Arabidopsis, a PP2C protein, HONSU, which acts as a negative regulator of ABA signaling, modulates the expression of GA metabolism and signaling genes and thereby enhance the transition of seeds from dormant to germinating state (Kim et al., 2013). In cereals such as rice, PP2C protein represses *OsbZIP10* (*ABI5* homolog of rice) via dephosphorylation and thereby promote seed germination (Bhatnagar et al., 2017). Consistently, overexpression of *OsPP2C51* leads to increased expression of α*-amylase* and activity of the corresponding enzyme. Since SbABI4 and SbABI5 from sorghum have been shown to interact with *SbGA2ox* promoter *in vitro* (Cantoro et al., 2013), the transcriptional repression of *OsbZIP10* (*ABI5*) by OsPP2C51 may imply decreased GA inactivation that leads to an increase in bioactive GA level and enhanced dormancy loss and germination. Furthermore, the expression of *HvPP2C* in the aleurone of barley appears to be differentially regulated by GA and ABA (Chen and An, 2006). All these results underline the significance of PP2C in mediating ABA and GA responses, thereby modulating the equilibrium between germination and dormancy; however, more studies are required to further elucidate this role of PP2C.

A recent study has also shown that Tiller Enhancer (TE) of rice, which acts as an activator of the APC/C^TE^ E3 ubiquitin ligase complex, mediates the balance between ABA and GA signaling and thereby the developmental switch of seeds between dormancy and germination (Lin et al., 2015; Shu et al., 2018). The interaction of TE with the ABA receptor OsPYLs/RCARs enhances proteasome mediated degradation of the receptor. However, ABA mediated activation of SnRK2s, which induces the phosphorylation of TE, ultimately represses the TE-OsPYLs/RCARs interaction and thereby stabilizes OsPYLs/RCARs, leading to increased seed sensitivity to ABA and enhanced dormancy. In contrast, GA enhances TE-OsPYLs/RCARs interaction and thereby the degradation of OsPYLs/RCARs through repression of the activity of SnRK2s, and this leads to decreased seed sensitivity to ABA and enhanced germination. Furthermore, a rice AP2-domain containing transcription factor, designated as OsAP2-39, regulates a switch between seed dormancy and germination through modulating the balance between ABA and GA levels via modulating the expression levels of *OsNCED1* and *Elongation of Uppermost Internode* (*EUI*) genes (Yaish et al., 2010; Shu et al., 2018). Studies in Arabidopsis have also shown that ABI4 regulates the equilibrium between seed dormancy and germination through modulating the balance between ABA and GA levels (Shu et al., 2013). For example, dry seeds of the *abi4* mutant of Arabidopsis contain lower and higher levels of ABA and GA, respectively, as compared to that of the wild-type. Consistently, ABI4 has been shown to enhance the expression of ABA biosynthetic (*NCED6*) and GA catabolic (*GA2ox7*) genes, although in a post-germination stage (Shu et al., 2016a), while repressing the expression of ABA catabolic (*CYP707A1* and *CYP707A2*) genes (Shu et al., 2013). However, further studies are needed to determine if a similar mechanism underlies the role of ABI4 in regulating seed dormancy and germination in cereals seeds.

**FIGURE 3:**
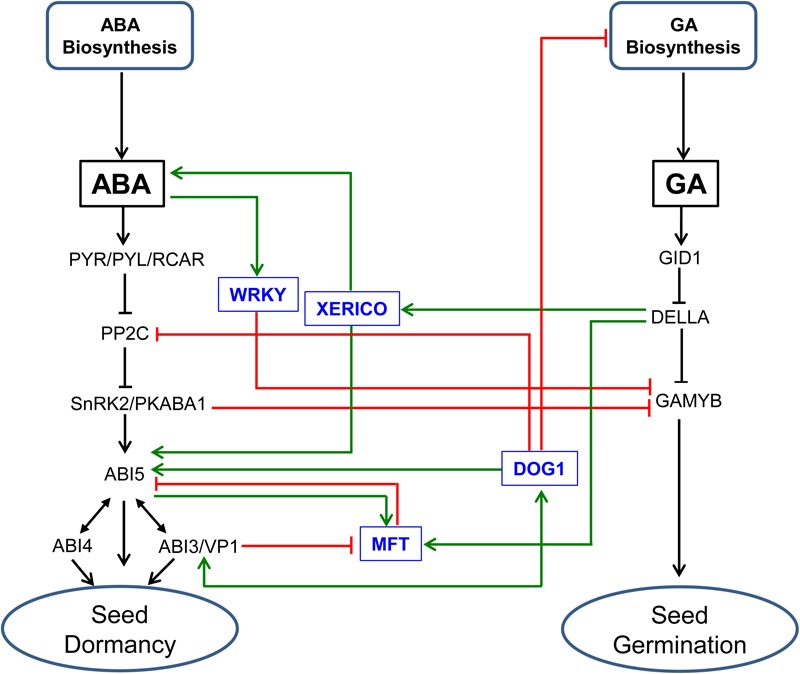
Genetic/molecular elements implicated in the regulation of ABA/GA balance and seed transition between dormancy and germination. ABA inhibits GAMYB-mediated GA responses through modulating the expression of WRKY transcription factors (Xie et al., 2006). DELLA protein regulates the balance between GA and ABA responses, and thereby seed dormancy and germination through its interaction with XERICO, a RING-H2 zinc finger E3 ubiquitin ligase. In the absence of GA, DELLA induces the expression of *XERICO*, which in turn enhances ABA accumulation, and ABI5 activity (Piskurewicz et al., 2008), leading to dormancy maintenance/inhibition of seed germination. *MOTHER OF FT AND TF1* (*MFT*) represses *ABI5* and therefore ABA signaling through a negative feedback mechanism (Xi et al., 2010). The role of MFT in regulating the balance between ABA and GA responses and thereby seed dormancy and germination involves ABI3 and ABI5 that act as repressor and activator of *MFT* expression, respectively, and DELLA that acts as activator of *MFT* expression (Xi et al., 2010). DELAY OF GERMINATION1 (DOG1) regulates ABA signaling and therefore seed dormancy through its interaction with PP2C (Née et al., 2017), and likely through modulating the expression of *ABI5* and interacting with ABI3 (Dekkers et al., 2016). DOG1 also regulates GA metabolism through the expressions of GA biosynthetic and inactivation genes in temperature dependent manner (Kendall et al., 2011; Graeber et al., 2014). SnRK2/PKABA1 binds to GAMYB and repress its transcription (Gómez-Cadenas et al., 2001). See the legends of **Figure 1** and **Figure 2** for descriptions of ABA and GA signaling components.

It has been shown that the DELLA proteins of Arabidopsis act as regulators of GA and ABA crosstalk and therefore the balance between dormancy and germination via its interaction with ABI3 and ABI5 (Lim et al., 2013). Furthermore, NUCLEAR FACTOR-YC (NF-YC) modulates GA and ABA signaling via the NF-YC–DELLA (RGL2)–ABI5 cascade independent of ABA (Liu et al., 2016); the NF-YCs binds to RGL2, which in turn regulates *ABI5*, a downstream component of ABA signaling, via specific CCAAT elements that are also shown to be present in the promoter of GA inducible *GAMYB* (Washio and Morikawa, 2006). The role of DELLA in mediating the balance between GA and ABA responses and in turn dormancy and germination can take place through its interaction with XERICO, a RING-H2 zinc finger E3 ubiquitin ligase that acts as important regulator of ABA signaling (Zentella et al., 2007; Piskurewicz et al., 2008) (**Figure 3**). When GA level is low, DELLA activates the expression of *XERICO*, which in turn enhances ABA accumulation and activates the transcription of *ABI5*, leading to dormancy maintenance/inhibition of seed germination. Consistently, overexpression of *XERICO* in rice results in increased ABA level and response via enhancing the expression of *OsNCED1* and *OsABI5*, respectively, causing a delay in germination (Zeng et al., 2014, 2015). Its overexpression in maize also leads to ABA accumulation via modulating the expression of *ZmCYP707A* rather than that of the *ZmNCED* genes as observed in rice (Brugière et al., 2017). However, detailed understanding of the role of XERICO in regulating the balance between ABA and GA response, and in turn seed transition between dormancy and germination awaits further study. It is well established that the germination of cereal seeds is characterized by transcriptional induction of hydrolytic enzymes such as α*-amylase*, which are required for the degradation of starch and its subsequent mobilization to the expanding embryo (Weiss and Ori, 2007). The expression of α*-amylase* in the aleurone of barley seeds can be activated by the GA inducible GAMYB (Gubler et al., 1995) but repressed by ABA induced PKABA1 (Ser-Thr kinase), which binds to GAMYB and represses its transcription (Gómez-Cadenas et al., 1999, 2001). However, RNAi mediated suppression of PKABA1 in barley does not affect ABA mediated repression of α-amylase in the seeds, suggesting the presence of PKABA1-independent ABA signaling pathway (Zentella et al., 2002). Indeed, a study in rice has shown that ABA mediated inhibition of GA inducible responses such as the expression of α*-amylase* can take place via an alternative pathway that involves WRKY transcription factors (Xie et al., 2006) (**Figure 3**).

Previous studies have also identified other genetic/molecular elements that are implicated in the regulation of seed dormancy and germination in cereals such as *MOTHER OF FT AND TFL1* (*MFT*) and *DELAY OF GERMINATION 1* (*DOG1*) (Ashikawa et al., 2010, 2014; Nakamura et al., 2011). Studies with the model plant Arabidopsis have demonstrated that MFT enhances seed transition from the state of dormancy to germination via feedback transcriptional repression of *ABI5* and ABA signaling (Xi et al., 2010). MFT also mediates ABA and GA responses, and this appears to involve the ABA signaling components ABI3 and ABI5 that act as repressor and activator of *MFT* expression, respectively, and the GA signaling component DELLA, which acts as transcriptional activator of *MFT* (Xi et al., 2010) (**Figure 3**). Recent reports have shown that DOG1 enhances ABA signaling and thereby seed dormancy through its interaction with PP2C (Née et al., 2017) and presumably by modulating the expression of *ABI5* and genetically interacting with ABI3 (Dekkers et al., 2016), implying that the action of DOG1 in regulating seed dormancy is coordinated with ABA (Nakabayashi et al., 2012). Mutational analysis of DOG1, furthermore, revealed that its role in dormancy maintenance is mediated at least partly by modulation of GA metabolism via altering the expressions of GA biosynthetic and inactivation genes in a temperature dependent manner (Kendall et al., 2011; Graeber et al., 2014) (**Figure 3**). However, it is yet unclear if the role of *MFT* and *DOG1* in the regulation of the developmental switch of cereal seeds between dormancy and germination is mediated by their interference with the balance between ABA and GA metabolism and signaling.

### Exogenous Factors Involved in the Regulation of ABA/GA Balance in Cereal Seeds

Seed imbibition environmental conditions such as temperature and light can also modulate the balance between seed dormancy and germination through altering the metabolic and signaling equilibrium between ABA and GA. Recent reports in wheat have demonstrated the role of imbibition temperature in regulating the balance of ABA/GA ratio and thereby seed transition between dormancy and germination (Kashiwakura et al., 2016; Izydorczyk et al., 2017). Imbibition of dormant seeds at lower temperature (15°C) induces germination through enhancing the expression of ABA catabolic (*TaABA8′OH1* and *TaABA8′OH2*) and GA biosynthesis (*TaGA3ox2*) genes that lead to a decrease in embryo ABA level while increasing that of GA (Kashiwakura et al., 2016). However, delay in germination as a result of seed imbibition at supraoptimal temperature (35°C) is mediated mainly by enhanced expression of ABA biosynthesis genes (*TaNCED1* and *TaNCED2*) that leads to increased ABA content in the embryo, and this ABA is also suggested to interfere with GA effects (Izydorczyk et al., 2017). Similarly, induction of dormancy/inhibition of germination as a result of imbibition at suboptimal temperature (4°C) appeared to be associated with increased expression of the *NCED* genes in both embryo and endosperm tissues and decreased expression of *CYP707A* genes in the endosperm, which ultimately led to increased seed ABA level. This effect of suboptimal temperature has been shown to be accompanied by repression of GA biosynthetic genes and GA levels in both embryo and endosperm tissues (Izydorczyk et al., 2017). The dynamic balance between ABA and GA signaling in regulating the switch of seeds between dormancy and germination can also be influenced by temperature. For example, the transition of wheat seeds from non-dormant/germinating to dormant state under supraoptimal imbibition temperature has been shown to be mediated by enhanced expression of embryonic genes that positively and negatively regulate ABA and GA signaling (Izydorczyk et al., 2017), respectively, leading to alteration of the balance between seed sensitivity to ABA and GA. In addition, seed imbibition at suboptimal temperature induces the expression of genes that regulate ABA signaling positively in both embryo and endosperm tissues, and this effect was accompanied by transcriptional activation of genes that negatively regulate GA signaling in the embryo.

Previous studies with the seeds of dicot species such as Arabidopsis have shown that red and far-red lights regulate dormancy and germination by altering the ABA/GA balance (Seo et al., 2006, 2009). The roles of these two lights in modulating the balance between ABA and GA metabolisms and therefore the transition of seeds between dormant and germinating states have also been investigated in monocot species. For example, the transition from dormant to germinating state of *Brachypodium* and photoblastic weedy rice seeds appeared to be promoted by red light but repressed by far-red light (Kim et al., 2009; Barrero et al., 2012), and these effects of the two lights in weedy rice seeds have been shown to be associated with changes in ABA and GA levels (Kim et al., 2009). Contrary to these observations, red and far-red lights are reported not to have an effect on the expression of ABA biosynthetic genes, the level of ABA and germination in barley seeds (Gubler et al., 2008). In addition to the two light qualities, previous studies investigated the roles of white and blue lights in regulation of seed dormancy and germination in cereals seeds. White and blue light are reported to inhibit the germination of barley and wheat seeds through increasing ABA content, and this appears to be mediated by transcriptional activation of the ABA biosynthetic *NCED* genes and repression of the ABA catabolic *ABA8*′*OH1* gene in the embryo (Gubler et al., 2008; Jacobsen et al., 2013; Hoang et al., 2014). Furthermore, the inhibitory effect of blue light but not that of white light on the germination of barley and rice seeds is associated with transcriptional repressions of GA biosynthetic gene and activation of GA inactivation genes (Gubler et al., 2008; Hirose et al., 2012; Hoang et al., 2014). Consistently, knocking-down of a rice gene encoding cryptochrome 1 (CRY1), which is a blue/ultraviolet-A photoreceptor, induces the expression of the GA inactivation *OsGA2ox* genes (Hirose et al., 2012). However, further mutational and genetic studies are required for detailed elucidation of the molecular mechanisms underlying the role of temperature and light in modulating ABA/GA balance and thereby developmental switch of seeds between dormancy and germination.

### Regulation of ABA/GA Balance in Cereal Seeds by Reactive Oxygen Species

Antagonistic interaction between ABA and GA and thereby the transition of seeds between dormancy and germination can also be mediated by ROS including hydrogen peroxide (H_2_O_2_), superoxide anion (O_2_^-^), and hydroxyl radical (OH^-^). The role of ROS in alleviating seed dormancy in cereals such as barley has been reported to be mediated mainly by the modulation of GA metabolism with almost no effect on ABA metabolism (Bahin et al., 2011). Consistently, ABA induced reduction of ROS production in rice seeds appeared to repress GA accumulation via repression of specific *OsGA20ox* and *OsGA3ox* genes, and thereby seed germination (Ye et al., 2012). Comparative analysis of dormant and non-dormant seeds revealed that the germination of non-dormant wheat and barley seeds is associated with the production of more ROS during imbibition (Caliskan and Cuming, 1998; Ishibashi et al., 2017), which in turn enhance the expression of ABA catabolic gene (*HvABA8*′*OH1*) and thereby reduce ABA level (Ishibashi et al., 2017). In agreement with these results, inhibition of the germination of non-dormant barley seeds through repression of ROS production has been shown to decrease embryonic GA level via repression of the expression of *TaGA20ox1* and *TaGA3ox1* genes, and increase ABA level via decreased expression of *HvABA8*′*OH1* (Ishibashi et al., 2015). Furthermore, GA induced promotion of H_2_O_2_ production in barley aleurone enhances the expression of α*-amylase* via activating the transcription of *GAMYB* and repressing the expression and activity of ABA inducible PKABA (Ishibashi et al., 2012). Despite these reports, the molecular bases underlying ROS mediated alteration of ABA/GA balance and the equilibrium between dormancy and germination in cereal seeds are still elusive.

## Conclusion and Future Perspectives

To date, considerable progress has been made in the dissection of molecular mechanisms underlying the control of ABA/GA balance and thereby seed transition between dormancy and germination in dicot species such as the model plant Arabidopsis. However, this phenomenon is still poorly understood in cereal seeds, and this emphasizes the need for further studies to determine if the molecular mechanisms identified in the seeds of dicot species are conserved in cereal seeds and identify novel mechanisms that are specific to cereal seeds. Although a limited number of molecular elements have been reported as regulators of ABA/GA interplay in cereal seeds, these elements are identified mainly based on comparative analysis of seeds from dormant and non-dormant lines or *in vitro* assays. Therefore, further genetic studies such as mutational and gain-of-function analyses are necessary to precisely pinpoint their physiological functions. Several studies have also provided important insights into the roles of other plant hormones, and genetic and epigenetic mechanisms in the control of dormancy and germination in cereal seeds. It is therefore interesting to explore if these factors influence dormancy status in cereal seeds through modulation of the ABA/GA balance. In recent years, there have been significant advances in the availability of genomic resources of cereal crops, and these resources are providing important platforms for the identification of molecular elements regulating ABA/GA balance in cereal seeds and elucidation of their roles in controlling seed transition between dormancy and germination. It is well established that preharvest sprouting, which causes substantial yield and quality losses in cereal crops, is closely associated with the level of dormancy manifested in the seeds. Therefore, a detailed understanding of molecular mechanisms underlying the regulation of ABA/GA balance and thereby dormancy and germination in cereal seeds will have a significant contribution in developing molecular/genomic tools that can enhance the breeding of cultivars with improved preharvest sprouting tolerance, and in turn lead to increased yield and quality of cereal crops.

## Author Contributions

BA conceived the topic and organized the manuscript. PAT, RK, and BA wrote and prepared the manuscript. PR and PKT were involved in the collection of literature and writing of Sections “ABA Signaling” and “Gibberellin Metabolism” of the manuscript.

## Conflict of Interest Statement

The authors declare that the research was conducted in the absence of any commercial or financial relationships that could be construed as a potential conflict of interest.
